# Relationship Between White Matter Lesions and Gray Matter Atrophy in Multiple Sclerosis: A Systematic Review

**DOI:** 10.1212/WNL.0000000000200006

**Published:** 2022-04-12

**Authors:** Ingrid Anne Lie, Merlin M. Weeda, Rozemarijn M. Mattiesing, Marijke A.E. Mol, Petra J.W. Pouwels, Frederik Barkhof, Øivind Torkildsen, Lars Bø, Kjell-Morten Myhr, Hugo Vrenken

**Affiliations:** From the Department of Radiology and Nuclear Medicine (I.A.L., M.M.W., R.M.M., P.J.W.P., F.B., H.V.), MS Center Amsterdam, Amsterdam Neuroscience, Amsterdam UMC, Location VUmc; Department of Clinical Medicine (I.A.L., Ø.T., L.B., K.-M.M.), University of Bergen, Norway; Medical Library (M.A.E.M.), Amsterdam UMC, Vrije Universiteit Amsterdam, the Netherlands; Institutes of Neurology and Healthcare Engineering (F.B.), UCL London, UK; Neuro-SysMed, Department of Neurology (I.A.L., Ø.T., L.B., K.-M.M.), Haukeland University Hospital, Bergen, Norway; Norwegian Multiple Sclerosis Competence Centre, Department of Neurology (L.B.), Haukeland University Hospital, Bergen, Norway.

## Abstract

**Background and Objectives:**

There is currently no consensus about the extent of gray matter (GM) atrophy that can be attributed to secondary changes after white matter (WM) lesions or the temporal and spatial relationships between the 2 phenomena. Elucidating this interplay will broaden the understanding of the combined inflammatory and neurodegenerative pathophysiology of multiple sclerosis (MS), and separating atrophic changes due to primary and secondary neurodegenerative mechanisms will then be pivotal to properly evaluate treatment effects, especially if these treatments target the different processes individually. To untangle these complex pathologic mechanisms, this systematic review provides an essential first step: an objective and comprehensive overview of the existing in vivo knowledge of the relationship between brain WM lesions and GM atrophy in patients diagnosed with MS. The overall aim was to clarify the extent to which WM lesions are associated with both global and regional GM atrophy and how this may differ in the different disease subtypes.

**Methods:**

We searched MEDLINE (through PubMed) and Embase for reports containing direct associations between brain GM and WM lesion measures obtained by conventional MRI sequences in patients with clinically isolated syndrome and MS. No restriction was applied for publication date. The quality and risk of bias in included studies were evaluated with the Quality Assessment Tool for observational cohort and cross-sectional studies (NIH, Bethesda, MA). Qualitative and descriptive analyses were performed.

**Results:**

A total of 90 articles were included. WM lesion volumes were related mostly to global, cortical and deep GM volumes, and those significant associations were almost without exception negative, indicating that higher WM lesion volumes were associated with lower GM volumes or lower cortical thicknesses. The most consistent relationship between WM lesions and GM atrophy was seen in early (relapsing) disease and less so in progressive MS.

**Discussion:**

The findings suggest that GM neurodegeneration is mostly secondary to damage in the WM during early disease stages while becoming more detached and dominated by other, possibly primary neurodegenerative disease mechanisms in progressive MS.

Gray matter (GM) atrophy occurs in patients with multiple sclerosis (MS)^e1^ already in early disease stages.^e2,1^ Reflecting axonal loss and irreversible neuronal damage,^2^ GM atrophy can be measured noninvasively in vivo from standard MRI. It is considered a marker of neurodegeneration that could help bridge the current gap between measures of clinical disability and traditional inflammatory MRI markers.^3^

Recent work has found that MS pathology affects both GM and white matter (WM) structures throughout the CNS. Therefore, it is unlikely that disability progression and worsening of higher functions such as cognition can be strongly predicted by a single MRI marker.^4^ Nevertheless, brain GM atrophy is associated with several clinical outcomes: GM volumes are lower in people with MS than in healthy controls,^5^ may predict conversion from clinically isolated syndrome (CIS) to MS,^6,7^ and relate to disability progression.^8^ Moreover, GM atrophy relates strongly with cognitive dysfunction^9-13^ and more so than WM lesion volume (LV).^14^

WM lesions have been the principal imaging marker of disease activity and progression in MS and are incorporated into diagnostic criteria^15^ and treatment goals,^16^ as well as outcome measures in research trials. These focal areas of demyelination, consisting of inflammation and variable gliosis,^17^ can be visualized as hyperintense or hypointense lesions in T2- and T1-weighted MRIs, respectively.^3^

If and how WM lesions and GM atrophy are temporally, spatially, and causally related are insufficiently clear. Elucidating this interplay will not only broaden understanding of the combined inflammatory and neurodegenerative pathophysiology of MS but also provide reliable biomarkers for research and therapeutic purposes. As treatment targets expand from inflammatory lesions to neurodegenerative processes, GM atrophy is a natural choice of outcome measure. Separating atrophic changes due to primary and secondary neurodegenerative mechanisms will then be crucial to properly evaluate treatment effects, especially if these treatments target the different processes individually. While some studies have addressed the relationship between WM lesions and GM atrophy directly, a larger body of literature reports measures of both. In this systematic review, we have therefore aimed to review this existing evidence in its entirety to establish how brain WM lesions and GM atrophy in MS are related.

## Methods

This review was conducted and presented according to the Preferred Reporting Items for Systematic Reviews and Meta-Analyses guidelines.^18^

### Search Strategy

To select studies of relevance to this systematic review, the electronic databases Medline (through PubMed) and Embase were searched. The search strategies were developed in consultation with a medical librarian (M.A.E.M.). Thesaurus terms and free-text words, including synonyms and closely related words, were used for the following concepts: MS, GM atrophy, and WM lesions. No restrictions were applied for language (at this stage) or publication date, but conference abstracts were excluded. The search strategy is detailed in eAppendix 1, links.lww.com/WNL/B816. The last search was conducted on August 17, 2020.

### Eligibility Criteria

Studies were included if they fulfilled all following criteria: (1) controlled trials or observational studies in English and published in a peer-reviewed journal; (2) trials or studies that involved patients diagnosed with CIS or MS; and (3) study abstract containing associations between brain GM and WM lesion measures obtained by conventional MRI sequences. To limit the scope of this review and the possible variability in pathologic substrates and disease mechanisms, we excluded studies of patients diagnosed with pediatric MS or with radiologically isolated syndrome.

### Outcome Measures

The primary outcome measures of interest were direct associations made between brain WM lesion and GM atrophy measures, obtained by conventional MRI sequences, in patients diagnosed with CIS or MS.

### Selection Process

After excluding duplicate publications, we screened the remaining abstracts on selection criteria by 2 independent raters (H.V., I.A.L.) using Rayyan software,^19^ a web-based application designed for systematic reviews.^20^ Conflicting selections were discussed until consensus.When eligibility could not be determined from the title and abstract alone, full texts of potentially relevant articles were consulted.

### Data Extraction and Quality Assessment

Independent extraction and quality assessment of relevant data from each included article were conducted by at least 2 reviewers (I.A.L., M.M.W., R.M.M., H.V.), according to a customized checklist. The quality and risk of bias in included studies were further evaluated with the Quality Assessment Tool for observational cohort and cross-sectional studies (NIH, Bethesda, MA). A rating scale of yes = 1, no = 0, and not reported = 0 was applied for the 14 questions of the checklist, and the final study quality was rated, in consensus between the raters (I.A.L. and H.V.), as good, fair, or poor on the basis of individual scores and the severity of the risk of bias.

To visually illustrate the main results for the different disease phenotypes, composite figures were prepared combining available, clear figures from key studies.

## Results

**Figure 1:**
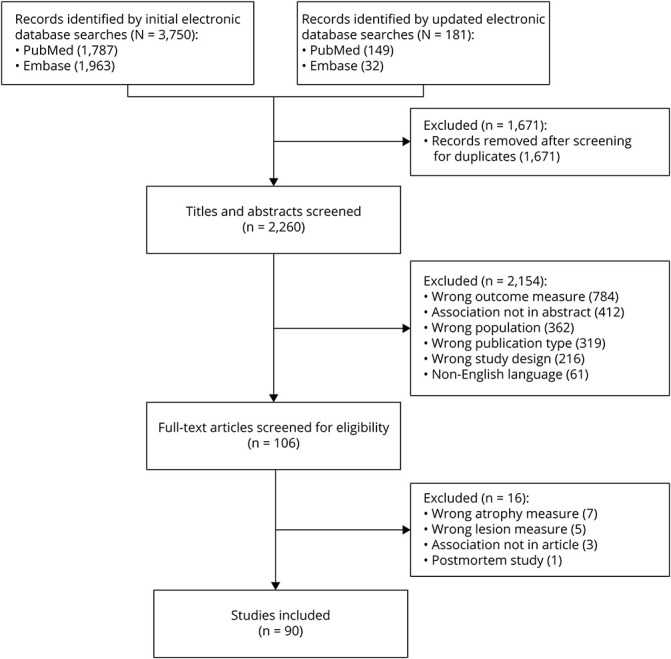
Flowchart Demonstrating the Selection Process

Through the initial search, 3,750 records were identified. After the updated search and removal of duplicates, 2,260 citations were screened on title and abstract, resulting in 106 full-text articles considered, of which 90 articles met the inclusion criteria and were included in this review (Figure 1). The 90 studies are listed in the eReferences (e1–e90, links.lww.com/WNL/B816), and the study design in all included articles is described in Table 1. Last, the quality assessment rate for each study is reported in eTables 1–3.

**Table 1:**
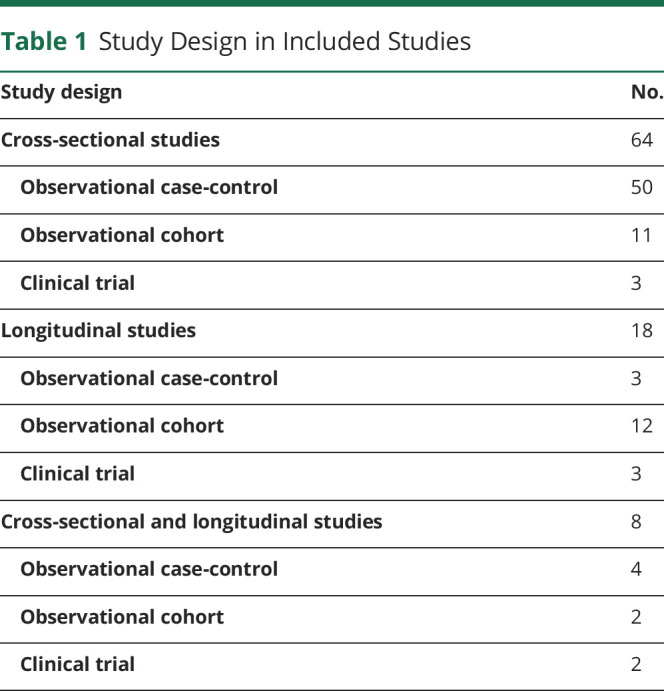
Study Design in Included Studies

### Clinically Isolated Syndrome

Eight cross-sectional and 4 longitudinal studies investigated patients diagnosed with CIS. In the longitudinal studies, the follow-up period ranged from 2 to 5.5 years.

The association of lesions with global GM measures were reported in 5 studies, while cortical GM (CGM) and deep GM (DGM) measures were each considered in 7 studies. Four studies reported regional WM lesion measures.

Included studies are described in eTables 1–3, links.lww.com/WNL/B816, and a more detailed discussion of results of each section is in eAppendix 2.

### Global GM in CIS

In 2 of 3 cross-sectional CIS studies, no significant association was found between global GM volume and either T2^e3^ or T1^e4^ LV. One study found a significant correlation between T2 LV and global GM volume (*r* = −0.56, *p* < 0.020).^e5^

The longitudinal relationship of global GM volume with global WM lesion measures was reported in 2 studies (follow-up time ranging from 2–3 years), both observing significant but different associations. In 1 study, change in global GM fraction correlated with WM LV changes (*r* values ranging from −0.3071 to −0.4280, *p* values from 0.0032 to 0.0426) but not with baseline lesion measures,^e6^ while the other study found associations with baseline lesion measures (*p* ≤ 0.004), but not with LV changes.^e7^

### CGM in CIS

In cross-sectional CIS studies, lower CGM volume showed variable associations with global WM lesion measures. Two studies observed a significant relationship with the presence (*t* = 2.48, *p* = 0.020)^e8^ or volume (*r* = −0.49, *p* = 0.045)^e5^ of T2 lesions, while 3 studies did not.^e2,e3,e8^ Of 2 studies reporting regional WM lesion measures, 1 study found a significant association between regional cortical thickness and T2 LV (*p* ≤ 0.0466),^e9^ while the other did not.^e8^

Of the 2 available longitudinal studies, 1 study found no relations,^e10^ while the other found significant associations of cortical volume change over 48 months with baseline WM lesion measures (*p* ≤ 0.004) and the total cumulative number of new/enlarging T2 lesions (*p* = 0.036), while no associations were observed for LV changes.^e7^

### DGM in CIS

In the 5 available cross-sectional studies in patients with CIS, all except 1 study^e3^ showed significant associations between global^e2,e4^ and regional^e11,e12^ WM LV and total^e2^ and regional DGM volumes^e2,e4,e11,e12^ (*p* values ranging from <0.0001–0.05). In contrast, no associations with DGM volumes were found for global T2 lesion number or the presence of gadolinium-enhancing lesions.^e2^ Of the regional DGM volumes investigated, the most consistent relationships were found for the thalamus and hippocampus. This pattern was true considering both global^e2,e4^ and regional WM LV.^e11,e12^

Longitudinally, 1 of the 3 available studies found that regional DGM atrophy was related to global baseline lesion measures (*p* ≤ 0.018),^e7^ but there was no relationship with changes in global^e7,e10^ or regional^e11^ LV (follow-up times between 2 and 5.5 years).

### Relapsing-Remitting MS

Overall, 37 cross-sectional and 14 longitudinal studies reported associations between WM lesion measures and GM atrophy in relapsing-remitting MS (RRMS). The follow-up period of the available longitudinal studies ranged from 1 to 5.5 years.

Seventeen publications reported the relationship with global GM, 29 on that with CGM, and 25 on that with DGM measures. Eleven studies considered regional WM lesion measures.

Included studies are described in eTables 1–3, links.lww.com/WNL/B816, and a more detailed discussion of results of each section is given in eAppendix 2.

**Figure 2:**
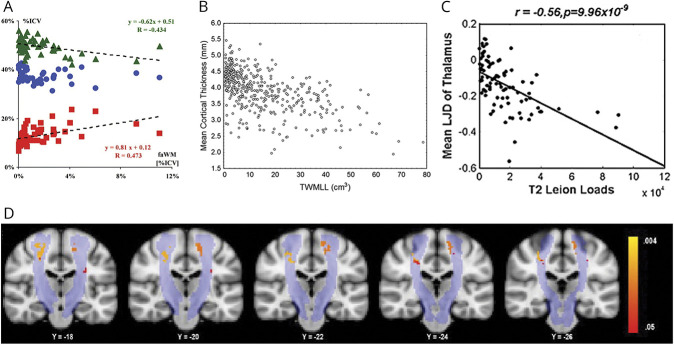
RRMS Shows Consistent Associations Between WM Lesions and GM Volume (A): Scatterplot of the fractional volumes of gray matter (fGM, green triangles), white matter (fWM, blue circles), and CSF (fCSF, red boxes) vs the fractional volume of abnormal white matter (faWM), all expressed as percentages of intracranial volume. fGM and fCSF values are adjusted to patients mean age (35.6 years). When significant, regression lines are shown, along with the corresponding equations and *R* values. Increasing loss of GM volume, with a corresponding increase in CSF volume, is apparent with increasing faWM. fWM (which includes also the white matter lesion volume) is not significantly changed with increasing faWM. Reproduced from Quarantelli et al., 2003,^e20^ with permission from Elsevier. (B) Scatterplot showing the relationship between mean cortical thickness in millimeters and total white matter lesion load (TWMLL) in cubic centimeters in 425 patients with relapsing-remitting multiple sclerosis (RRMS). Reproduced from Charil et al., 2007,^e35^ with permission from Elsevier. (C) Correlation of the mean logarithm of the jacobian determinant (LJD; a measure of atrophy) with T2 lesion load in 88 patients with RRMS for the thalamus. Reproduced from Tao et al., 2009.^e37^ with permission from Elsevier. (D) Lesional voxels that significantly correlate with primary motor cortex thickness are shown in red-yellow. Probabilistic corticospinal tract atlas is shown in light blue. Reproduced from Bergsland et al., 2015,^e47^ with permission from SAGE Publications.

Figure 2 illustrates the main results from this section.

### Global GM in RRMS

The majority of available cross-sectional RRMS studies, i.e., 8 of 10 studies, observed significant associations between global GM volume and global WM lesion load. Eight studies observed a significant association between global GM volumes and T1^e13,e14^ and T2 LV^e13-e19^ and abnormal WM^e20^ (*p* values ranging from <0.001–0.047). In contrast, 2 studies considering T2 LV^e21,e22^ and another 2 studies considering gadolinium-enhancing LV^e13,e14^ did not.

One cross-sectional study investigated the impact of regional LV on total GM volume and reported a significant correlation with regional T1 and T2 LV in 3 and 4 of 26 WM regions, respectively (*r* values ranging from −0.20 to −0.50, *p* < 0.001).^e23^

Of the 7 longitudinal studies available, 4 did not find an association between global GM atrophy progression and global WM lesion measures. When considering gadolinium-enhancing lesion measures obtained at baseline, 1 study found a significant association (*p* = 0.04),^e24^ while 3 others did not find that global GM atrophy progression related to the presence,^e25,e26^ number,^e26^ or volume^e14^ of gadolinium-enhancing lesions (follow-up time ranging from 1–4 years).

Three of 5 studies considering longitudinal WM lesion changes^e14,e24,e27-e29^ with a follow-up period between 1 and 4 years observed significant associations between longitudinal changes in T1^e27^ and T2^e24,e27,e28^ LV and GM atrophy progression (*p* values ranging from 0.0004–0.03).

### CGM in RRMS

A majority of cross-sectional studies (14 of 19) considering global WM LV found significant associations. WM LV was found to relate negatively to both total cortical volume (*p* values ranging from <0.0001–0.05)^e2,e15,e30-e32^ and global cortical thickness (*p* values ranging from <0.001–<0.05).^e17,e30,e33-e35^

A total of 6 studies explored global T1^e36,e37^ and T2^e16,e19,e30,e36-e38^ lesions and their relationship with regional cortical volume, with the most consistent and strongest associations in areas in the frontal, temporal, cingulate, and insular cortex. A similar pattern of associations was seen for cortical thickness measures.^e30,e35,e39^

Five studies did not find significant relations for either cortical volume^e21,e40-e42^ or cortical thickness.^e39^

In 6 of the 8 cross-sectional studies^e9,e23,e35,e43-e47^ considering regional distribution of WM lesions, the results suggested an anatomic or structural relationship between lesion location and regional cortical volume^e44,e45^ and thickness.^e9,e35,e46,e47^

Considering global WM lesion measures, 3 of five^e10,e27,e32,e48,e49^ longitudinal studies found significant relationships between both baseline WM lesion measures^e49^ and on-study changes in WM LV^e27,e48^ or numbers^e48^ and cortical thinning (*p* = 0.040),^e48^ as well as regional (*p* < 0.01)^e27^ and total cortical volume loss (*p* values ranging from <0.0001–0.010)^e49^ (follow-up time ranging from 1–2 years).

Of the 2 studies assessing regional WM LV, 1 study observed visually that the increase in T2 LV spatially coincided with areas of cortical decrease,^e50^ while the other study did not.^e51^

### DGM in RRMS

With the exception of 1 study,^e52^ all 17 cross-sectional publications reporting global WM LV found significant associations with DGM volume measures. Three studies evaluated DGM volume as a whole (*p* values ranging from <0.0001–0.04),^e17,e49,e53^ while the remaining assessed the various structures separately. Thalamic volume and surface displacement^e54^ were associated negatively with T1^e36,e37^ and T2^e2,e16,e30,e36-e38,e40,e41,e49,e54,e55^ LV in 11 studies (*p* values ranging from <0.00001–<0.05). Other DGM structures repeatedly showing significant associations with WM LV were the caudate nucleus (*p* values ranging from <0.0001–<0.05),^e2,e19,e36-e38,e41,e42,e55,e56^ putamen (*p* values ranging from <0.00001–<0.05),^e2,e30,e36-e38,e53,e55^ and globus pallidus (*p* values ranging from <0.0001–<0.05).^e2,e30,e38,e54,e55^

While 2 cross-sectional studies did not find any associations between regional WM lesion and DGM measures,^e23,e45^ the majority of studies did.^e43,e44,e46,e54^

All 4 publications that assessed longitudinal relations between total and regional DGM atrophy and global WM lesion measures observed significant associations.^e10,e48,e49,e57^ The associations were found for both baseline WM lesion measures (*p* < 0.0001 and 0.037)^e49,e57^ and on-study new/enlarging T2 lesions or new gadolinium-enhancing lesions (*p* = 0.024).^e48^

### Secondary Progressive MS

Eleven cross-sectional and 2 longitudinal studies reported patients with secondary progressive MS (SPMS), 5 of which explored associations between WM lesions and global GM volume, while 8 and 10 studies focused on CGM and DGM measures, respectively. Four studies considered regional WM lesion measures.

Included studies are described in eTables 1 and 2, links.lww.com/WNL/B816, and a more detailed discussion of results of each section is given in eAppendix 2.

**Figure 3:**
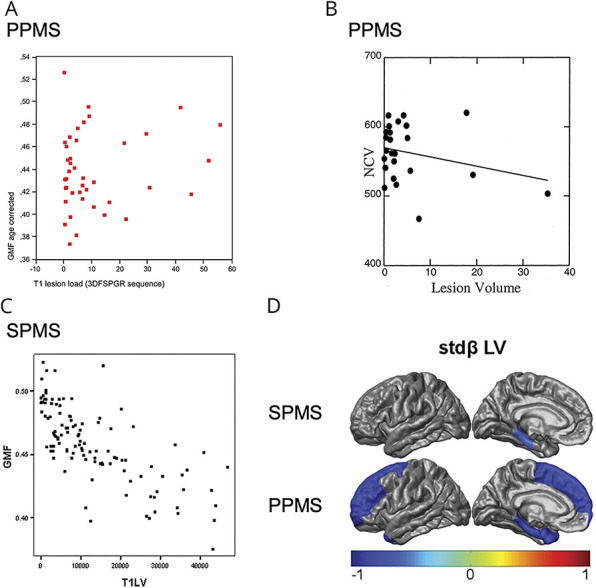
Progressive MS Shows Varying Associations Between WM Lesions and GM Volume (A) Primary progressive multiple sclerosis (PPMS): In 43 patients with PPMS, gray matter (GM) fraction (GMF) corrected for age is plotted against T1 lesion load (in milliliters; derived from 3-dimensional fast spoiled gradient recalled echo scans), illustrating an absence of correlation. Reproduced from Sastre-Garriga et al., 2004,^e59^ with permission from Elsevier. (B) PPMS: data illustrating the absence of correlation between normalized cortical volume and T2 lesion volume in 25 patients with PPMS (Spearman rank coefficient *r* = −0.1, *p* = 0.6). Reproduced from De Stefano et al., 2003,^e31^ with permission from Wolters Kluwer Health, Inc. (C) Secondary progressive multiple sclerosis (SPMS): scatterplot of T1 lesion volume (T1LV) vs GMF in 117 patients with SPMS, illustrating a significant correlation (*r* = −0.72, *p* < 0.001). Reproduced from Furby et al., 2009,^e58^ with permission from SAGE Publications. (D) Graphic visualization of the cross-sectional relationship between regional cortical thickness and white matter (WM) measures in 53 patients with long-standing SPMS (top row) and 25 patients with longstanding PPMS (bottom row), as assessed through linear regression. In gray areas, lesion volume in the connected WM tracts did not contribute significantly to the model explaining regional cortical thickness, whereas in colored areas, colors correspond to the standardized beta values of lesion volume in the connected tracts for the respective regional model. Reproduced from Steenwijk et al., 2015,^e46^ with permission from John Wiley and Sons.

Figure 3 illustrates the main results from this section.

### Global GM in SPMS

Three of 4 cross-sectional studies reported negative associations between WM LV and global GM volume (*r* values ranging from −0.36 to −0.72, *p* values ranging from <0.001–<0.01).^e16,e17,e58^ The 1 study considering regional T1 and T2 LV and global GM volume found no significant associations.^e23^

Longitudinally, neither baseline nor on-study changes in WM lesion measures predicted changes in GM volume over the 4-year follow-up.^e24^

### CGM in SPMS

The observed relationship between cortical volume or thickness and global WM lesion load in patients with SPMS was not consistent in the 4 available studies. Two studies found significant associations with lower cortical volume (*p* < 0.001 and <0.05).^e16,e40^ Furthermore, cortical thickness was evaluated in another 2 studies based on 1 and the same study population; neither study found any significant association between T2 LV and global mean cortical thickness.^e17,e34^

One of 4 cross-sectional studies assessing regional WM lesions observed relatively strong correlations between lower cortical volume and T2 LV in the same or adjacent lobes (*r* values ranging from −0.67 to −0.79, *p* < 0.001).^e40^ In the 3 remaining studies, the associations with lower cortical volume or thickness were weak^e45,e46^ or nonsignificant.^e23^

The only longitudinal study available investigated atrophied T2 LV (T2-weighted lesional tissue subsequently substituted by CSF) in patients with SPMS and primary progressive MS (PPMS) in a combined progressive MS group, finding no associations with baseline cortical volume or volume change (follow-up time 5.5 years).^e10^

### DGM in SPMS

In the 6 cross-sectional publications that considered global WM lesions, results were somewhat conflicting. Two studies found no associations with lower DGM volume^e16,e55^; in the other 4 studies, however, T1^e53^ or T2^e17^ LV was associated significantly with both total DGM volume (*p* values ranging from <0.01–0.04) and separate DGM structures such as the hippocampus,^e52^ thalamus, and caudate nucleus^e40^ (*r* values ranging from −0.69 to −0.88, *p* < 0.001–0.018). Two of 4 studies^e23,e40,e45,e46^ that included results for regional WM LV or distribution showed significant associations^e40,e46^ (*p* values ranging from <0.001–<0.05).

The sole longitudinal study found no association between atrophied T2 LV (described in the previous section) and baseline thalamic volume or volume change.^e10^

### Primary Progressive MS

The relationship between WM lesions and GM measures in patients with PPMS was assessed in 11 cross-sectional studies and longitudinally in 2 studies.

Three studies performed analyses involving global GM volume, while CGM and DGM measures were each considered in 9 studies. Three studies considered regional WM LV or distribution.

Included studies are described in eTables 1 and 2, links.lww.com/WNL/B816, and a more detailed version of the respective sections is given in eAppendix 2.

Figure 3 illustrates the main results from this section.

### Global GM in PPMS

The cross-sectional associations between global WM lesion measures and global GM volume in patients with PPMS were variable. One study reported a significant correlation with T2 LV (*r* = −0.68, *p* < 0.001),^e17^ while the other found no significant associations with either T2, T1, or gadolinium-enhancing LV or lesion numbers.^e59^

In the available longitudinal study, baseline WM lesion measures were not related to GM volume change over 12 months.^e60^

### CGM in PPMS

Cross-sectional results for global WM lesion measures and CGM volume or thickness in patients with PPMS were divided. Three studies found associations between T1^e61^ and T2^e34,e40^ LV and total (*r* = −0.508, *p* < 0.05)^e61^ and regional (*r* values ranging from −0.605 to −0.85, *p* values ranging from <0.001–<0.01)^e40^ cortical volume and total cortical thickness (*p* < 0.05).^e34^ In the other 3 studies, no significant associations were found for either cortical volume^e31,e62^ or thickness.^e17^

Of 3 publications assessing regional WM lesion measures, 1 study found a relationship with cortical volume in anatomically connected areas (*p* < 0.001),^e40^ while in the other 2, the associations with cortical thickness or volume were weak^e46^ or absent.^e45^

Only 1 longitudinal study was identified, finding no associations between atrophied T2 LV (described in previous section) and baseline cortical volume or volume change.^e10^

### DGM in PPMS

All but 1^e55^ of the 6 cross-sectional studies reporting the relationship between global WM lesions and DGM volume observed significant associations. In patients with PPMS, correlations were significant for both total DGM volume (*r* values ranging from −0.651 to −0.71, *p* values ranging from <0.001–<0.01)^e17,e61^ and the separate structures. The most consistent association with global WM LV was seen for the thalamus^e62^ for both T2 (*r* values ranging from −0.48 to −0.94, *p* values ranging from <0.001–<0.05)^e40,e61,e63^ and T1 (*r* values ranging from −0.44 to −0.554, *p* values ranging from 0.002–<0.05)^e61,e63^ LV.

Of 3 cross-sectional publications assessing regional WM lesions,^e40,e45,e46^ lower regional DGM volume was related to regional T2 LV in 2 studies (*p* values ranging from <0.001–<0.05).^e40,e46^

Again, only 1 longitudinal study was available, and for both baseline thalamic volume and volume change, no relationship to atrophied T2 LV (described in the previous section) was found.^e10^

### Results for Mixed MS Groups

A number of studies^e1,e9,e10,e16,e17,e23,e34,e46,e53,e55,e64-e90^ reported analyses relating GM atrophy measures to WM lesion measures in heterogeneous groups of patients with MS encompassing different disease phenotypes. Full results of these studies are reported in eAppendix 2, links.lww.com/WNL/B816. Briefly, in most cross-sectional studies, GM atrophy and WM lesions were significantly associated; in longitudinal studies, results were more variable.

### Comparisons Between Disease Phenotypes

Some of the studies discussed in the previous sections included multiple disease phenotypes in a single study. Such a design eliminates differences between image acquisition and image analysis approaches that may otherwise account for differences between disease phenotypes observed from separate studies and therefore can shed the most direct light on whether the relationship between GM atrophy and WM lesions might differ between disease types. Table 2 summarizes the observed associations in articles including multiple phenotypes, and full reports of the studies are given in eAppendix 2, links.lww.com/WNL/B816. We focus on whether the observations differed between disease types and, when available, on the direct statistical comparisons between disease types. In summary, for global GM, CGM, and DGM, both cross-sectional and longitudinal studies found the most consistent associations with WM lesions in RRMS, while the associations for CIS, SPMS, and PPMS were more variable (Table 2). In 11 of 15 studies, the largest patient group consisted of patients with RRMS, often in a great majority. Such imbalance may cause the studies to detect significant associations only in the larger patient group, merely because of power and not due to a lack of true association in the smaller (progressive) patient group.

**Table 2:**
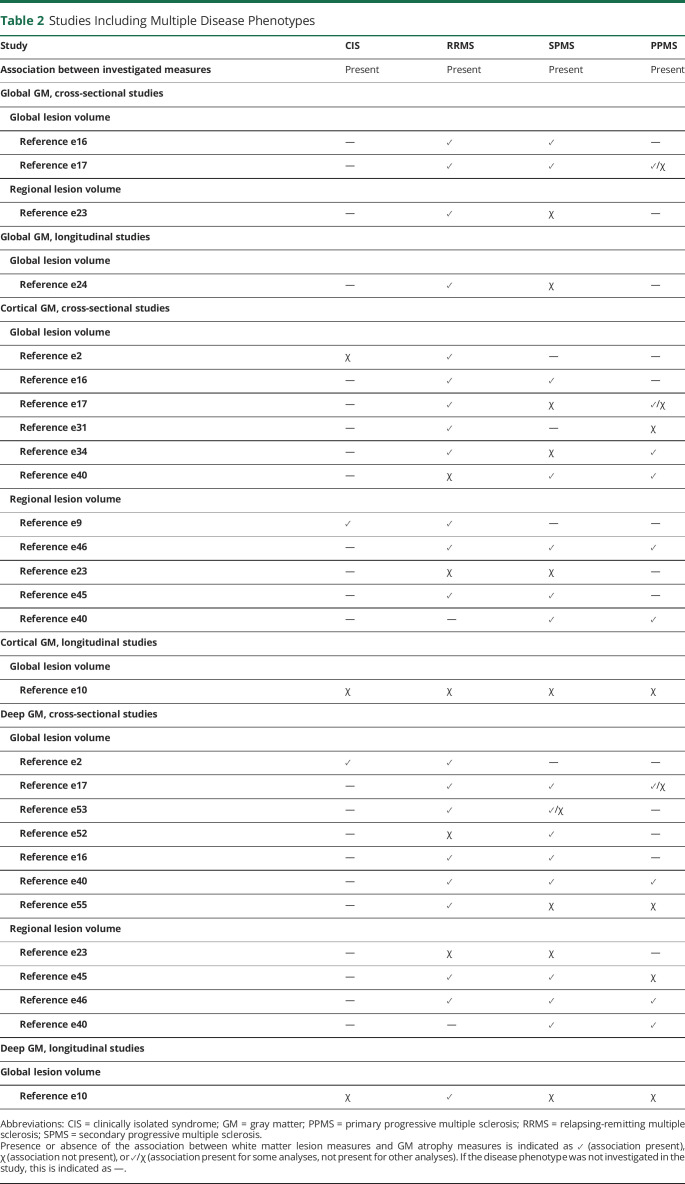
Studies Including Multiple Disease Phenotypes

The included studies are described in eTables 1 and 2, links.lww.com/WNL/B816.

## Discussion

This systematic review assessed the existing evidence regarding an association between brain WM lesions and GM atrophy in MS. Surveying results from cross-sectional and longitudinal studies of different phenotypes and with varying anatomic regions of interest has resulted in a comprehensive picture. More WM lesions were associated with more GM atrophy (Table 3), especially in RRMS and less consistently so in progressive MS.

**Table 3:**
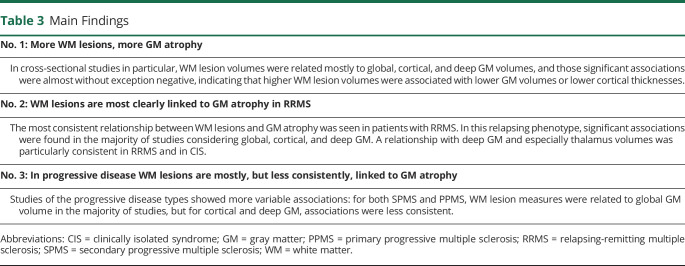
Main Findings

The quality of evidence was mostly rated as fair, with no correction for potential confounders (e.g., therapeutic and physiologic factors), short follow-up time, and small or unbalanced disease groups (as highlighted in the previous section) as the main risks of bias.

The clear trend emerging from cross-sectional and longitudinal studies for both global and regional associations was that more WM lesions were related to more or faster GM atrophy. Patients with high WM lesion burden may be expected to also have extensive damage to other brain structures, not necessarily because one causes the other but possibly also because an advanced disease stage acts as a common denominator. To further investigate, each disease type was evaluated and compared; the association was observed frequently in all disease types, most consistently in RRMS. However, the relationship was more variable for longitudinal than for cross-sectional outcomes.

In mixed MS groups, the lack of significant associations in longitudinal studies could be related to group heterogeneity. Furthermore, variable treatment regimens across patients affect the interpretation of all studies, especially more recent longitudinal studies. Here, the time that patients have spent under potent treatment is often considerable and may modulate not only the observed association between WM lesions and GM atrophy but also the main pathologic substrate of the neurodegenerative process.

Current knowledge from neuroimaging and histopathology implies that GM neurodegeneration is driven both by events secondary to WM inflammation and by primary disease mechanisms within the GM. Adding to the complexity, these mechanisms seem to act simultaneously, with additive effects.^21^ Strong and consistent associations with WM lesions were found in all GM regions in RRMS and in DGM in CIS; this suggests that early GM neurodegeneration is mainly secondary to damage in the WM: after chronic inflammation in WM, neuronal injury and damage to mitochondria with resulting energy deficiency initiate several neurodegenerative cascades. The degenerative process can move forward toward the axonal terminal (anterograde or wallerian degeneration) or backward toward the cell soma (retrograde degeneration), leading to neuronal loss and atrophy in connected GM regions.^22^ In both CIS and RRMS, the most consistent relations were seen in DGM and the thalamus. Connecting and relaying information between subcortical areas and the neocortex through different WM tracts,^23^ it seems plausible that thalamic GM components are vulnerable to damage through retrograde degeneration.^24^

In progressive MS phenotypes, while GM atrophy was more widespread, affecting most DGM structures^e40,e46,e55^ and cortical areas,^e40,e46^ the relationship with focal WM lesions was more varied although still present in the majority of studies.^e17,e34,e46,e55^ These results, interpreted together with neuropathologic studies showing continued, widespread GM atrophy development, at least partly independently of focal inflammatory WM lesions,^25-27^ suggest that in progressive MS the neurodegenerative disease mechanism may be a mainly primary process. Alternatively, in long-standing MS with many tracts affected, the relationships between primary lesional damage and downstream GM atrophy may become too complex and too variable across individuals to disentangle. Furthermore, GM lesions, often found more prominently in progressive MS, may also propagate GM atrophy and contribute to its less consistent association with WM lesions.^e53^ In addition, consistent GM atrophy patterns found in patients with CIS^e70^ suggest that some primary degenerative processes may be present throughout the disease.

The reviewed literature suggests that the mechanisms of neurodegeneration in MS are not static through the disease course, so the therapeutic targets, interventions, and subsequent monitoring will most likely differ for the various patient groups. To obtain fully individualized and optimized patient treatment, we have summarized important research aims and suggestions for future research in Table 4.

**Table 4:**
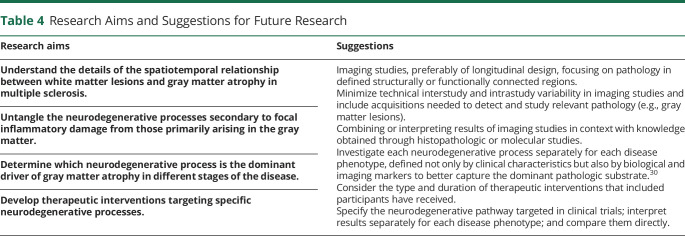
Research Aims and Suggestions for Future Research

Our study has several limitations. Diagnostic criteria and hence the separation between CIS and MS varied over time. The effect of physiologic variability and therapeutic interventions was not consistently accounted for in the reviewed articles. Whether treatment was used and what type were mostly stated but rarely adjusted for in the analyses. Therefore, effects of individual treatments on WM lesions or (primary or secondary) GM atrophy, which potentially change the observed relationship between the 2 processes for each patient, could cloud our interpretation of the disease mechanisms.

In longitudinal studies, the group sizes were often smaller, and the majority followed up the patients for ≤2 years. Such short follow-up durations most likely affected the ability to detect temporal associations, considering that neurodegeneration is a slowly progressive process.^e24^ Moreover, brain atrophy is cumulative and may exhibit a ceiling effect and delayed effects from previous exposures or previous pathologic damage.^28^

Technical factors are well known to affect brain measurements^28^: intrastudy and interstudy variability in MRI scanners and acquisitions (e.g., field strength, slice thickness, 2-/3-dimensional acquisitions, pulse sequence type and parameters), image (pre)processing tools, and analysis software. This makes the interpretation and comparison of results challenging. The 20-year time frame of included articles, during which MRI technology has achieved major leaps of improvement, means that earlier studies have to be evaluated in the light of the concurrently available technology and knowledge. Furthermore, the large variability in image acquisition, analysis methods, and outcome measures, combined with uncertainties about potential confounders such as treatment, made it impossible to conduct a meaningful and interpretable meta-analysis of the results reported in the reviewed articles.

Statistical issues may also have influenced results. Sample sizes were often unbalanced between disease types, especially with small progressive groups being compared to larger relapsing-remitting groups. Furthermore, the majority of studies focused on patients with RRMS, which limits our ability to draw conclusions for progressive disease types.

To elucidate the pathophysiologic relationship between inflammatory WM lesions and neurodegenerative changes in GM, this review has an obvious limitation in that statistical associations do not prove causation. However, the many imaging studies included provide the possibility to investigate these relations in vivo in a large number of patients in different disease stages. Although in this study a spatiotemporal relationship between changes in GM structures and WM lesions was found, we cannot draw any conclusions about whether this process starts with demyelination in WM or whether the primary defect is in the axon or neuron itself, with demyelination as a secondary effect.^29^ To widen this question of causality, some researchers suggest that the association seen between WM lesions and lower GM volume in certain regions is not causally linked through axonal degradation but is mainly due to a common close proximity to inflammatory soluble factors in the CSF.^e9^

Due to capacity and limiting the scope of this systematic review, we included only MRI measures obtained by conventional MRI sequences. Advanced imaging methods would be interesting to review, which by necessity would require more attention to the myriad technical differences between such studies.

We found that the majority of the literature overwhelmingly reported an association between WM lesions and global or regional GM atrophy. The association was most consistent in RRMS but more variable in progressive phenotypes and CIS. This suggests that GM neurodegeneration is mostly secondary to damage in the WM during early disease stages, while more detached and dominated by other, possibly primary neurodegenerative disease mechanisms in progressive MS.

These findings are of great importance for patient treatment and research, indicating that the most effective targets for neuroprotective treatment change throughout the disease course.

To further disentangle the secondary GM atrophy caused by WM damage from primary neurodegenerative disease mechanisms, more studies investigating the spatiotemporal relationship between the 2 pathologic phenomena are needed, preferably with extensive follow-up time and a direct comparison with the different disease phenotypes.

## Glossary

CGM: cortical GM
CIS: clinically isolated syndrome
DGM: deep GM
GM: gray matter
LV: lesion volume
MS: multiple sclerosis
PPMS: primary progressive MS
RRMS: relapsing-remitting MS
SPMS: secondary progressive MS
WM: white matter
